# Editorial

**Published:** 1867-10

**Authors:** 


### Editorial.

Chicago Medical College.—The Ninth Annual Course of Instruction in the Chicago Medical College, will commence on Monday, Oct. 7th, 1867. The general introductory to the course will be given in the College Hall on Monday evening, at 7½ o'clock, by D. J. Nelson, M.D., Professor of Physiology and Histology. The College building has been put in good repair, and the lecture rooms re-seated in excellent style. A good class have been in attendance on the clinical instruction in Mercy Hospital all the past month; and there is a good prospect of a full class in the College.

Rush Medical College.—The opening of the regular College Term in the new building of Rush Medical College, is announced to take place on Wednesday, Oct. 2d, 1867.

Chicago Medical Society.—The regular meetings of this Society are now being held on Friday evening of each week, in the County Court-Room. The last meeting was well attended, and chiefly occupied in the discussion of the therapeutic value of subcutaneous injections. The facts elicited during the discussion seemed to show that the usual effects of any of the alkaloids could be very promptly and efficiently induced by this method, and with from twenty-five to fifty per cent less than would be required for administration by the mouth; and that pure aqueous solutions of these substances seldom induced any local inflammation in the tissues into which they were injected. But other facts seemed to show that the subcutaneous use of solutions containing any of the mineral acids in a free state, lactic acid, or lactic acid salts, often induced inflammation, and sometimes extensive ulceration or destruction of the areolar tissue.

---

Public Health.—The general health of this city is good; no epidemic of any kind having occurred during the past summer.

---

Medical Books.—The very extensive bookselling house of S. C. Griggs & Co., have laid on our table a neatly-printed catalogue of their Medical Books, with prices attached. It presents a very extensive list, embracing almost every work likely to be wanted by either student or practitioner. These catalogues are furnished to all applicants, free of expense.

---

Apology.—The article from Dr. Brickett, on the Use of Chloroform in the Treatment of Intermittent Fever, was received several months since, but was accidentally overlooked until the present time.

---

Chicago Charitable Eye and Ear Clinic—504 State St. Open daily at 12 o'clock, for the gratuitous treatment of diseases and defects of the eye and ear.

OFFICERS:

*Board of Trustees.*—Hon. Lyman Trumbull, President;

Hon. C. N. Holden, Wm. E. Doggett, Esq., C. T. Bowen, Esq., P. L. Yoe, Esq., Secretary and Treasurer.

*Attending Surgeon.*—Joseph S. Hildreth.

*Dispensing Chemist.*—Colin Groff.

This institution having become fully established, the Trustees are desirous of making it more generally known.

Its benefits are not limited to the city, but available to all whose circumstances entitle them to such assistance.

The Surgeon will be in attendance from 12 to 1 o'clock every day in the week, except Sunday, at the Consultation Rooms, No. 504 State Street, corner of Taylor.

---

The following circular has been addressed to the Faculties of all the Medical Colleges, known to exist at present, in the United States. We commend the object had in view, and the plan proposed, to the careful consideration of the whole profession.—[Editor.

TO MEDICAL COLLEGES.

---

At the Convention of Delegates from Medical Colleges, called for the purpose of revising the system of Medical College instruction in this country, and which convened in Cincinnati, May 3d, 1867, the following resolution was unanimously adopted:

"*Resolved*, That a committee of five be appointed by the President, whose duty it shall be to present the several propositions adopted by this Convention, to the Trustees and Faculties of all the Medical Colleges in this country, and solicit their definite action thereon, *with a view to the early and simultaneous practical adoption* of the same throughout the whole country. And that the same committee be authorized to call another convention whenever deemed advisable."

The undersigned Committee, appointed for the purpose of carrying into effect the instructions contained in the foregoing resolution, respectfully invite the attention of the Trustees and Faculty of each duly organized Medical College in the United States, to the five following propositions, which, after mature deliberation, were adopted by the said Convention with entire unanimity:—

"*Resolved*, 1st. That every student applying for matriculation in a Medical College shall be required to show, either by satisfactory certificate, or by direct examination by a committee of the Faculty, that he possesses a knowledge of the common English branches of education, including the first series of mathematics, the elements of the natural sciences, and a sufficient knowledge of Latin and Greek to understand the technical terms of the profession; and that the certificate presented, or the result of the examination thus required, be regularly filed as a part of the records of each Medical College.

"2d. That every medical student shall be required to study four full years, including three regular annual courses of medical college instruction, before being admitted to an examination for the degree of Doctor of Medicine.

"3d. That the minimum duration of a regular annual lecture term, or course of medical college instruction, shall be six calendar months.

"4th. That every Medical College shall embrace in its Curriculum the following branches, to be taught by not less than nine Professors, viz.:—

"Descriptive Anatomy, including dissections; Physioloy and Histology; Inorganic Chemistry; Materia Medica; Organic Chemistry and Toxicology; General Pathology, Therapeutics, Pathological Anatomy, and Public Hygiene; Surgical Anatomy and operations of Surgery; Medical Jurisprudence and Medical Ethics; Practice of Medicine; Practice of Surgery; Obstetrics, and Diseases of Women and Children; Clinical Medicine, and Clinical Surgery; and that these several branches shall be divided into three groups or series, corresponding with the three courses of Medical College instruction required.

"The first, or *Freshmen series*, shall embrace Descriptive Anatomy and Practical Dissections; Physiology and Histology; Inorganic Chemistry, and Materia Medica. To these the attention of the student shall be mainly restricted during his first course of Medical College instruction, and in these he shall submit to a thorough examination by the proper members of the Faculty, at its close, and receive a certificate indicating the degree of his progress.

"The second, or *Junior series*, shall embrace Organic Chemistry and Toxicology; General Pathology, Pathological Anatomy, Therapeutics, and Public Hygiene; Surgical Anatomy and operations of Surgery; Medical Jurisprudence and Medical Ethics. To these the attention of the medical student shall be directed during his second course of Medical College instruction, and in them he shall be examined at the close of his second course, in the same manner as after the first.

"The third, or *Senior series*, shall embrace Practical Medicine; Practical Surgery; Obstetrics and Diseases peculiar to Women and Children; with Clinical Medicine and Clinical Surgery in a hospital. These shall occupy the attention of the student during his third course of college instruction, and at its close he shall be eligible to a general examination for the degree of Doctor of Medicine.

"The instruction in the three series is to be given simultaneously, and to continue throughout the whole of each annual college term; each student attending the lectures on such branches as belong to his period of progress in study, in the same manner as the sophomore, junior, and senior classes, each pursue their studies simultaneously throughout the Collegiate year in all our Literary Colleges.

"5th. That every Medical College should immediately adopt some effectual method of ascertaining the actual attendance of students, upon its lectures and other exercises, and at the close of each session, or of the attendance of the student, a certificate specifying the time and the courses of instruction actually attended, should be given, and such certificate *only* should be received by other colleges as evidence of such attendance."

It will be seen that these propositions are designed to introduce into the system of Medical College instruction in this country, *four changes* of great practical importance, namely:—1st. A positive standard of preliminary education. 2d. A longer time in which to acquire a knowledge of the various branches of Medical Science and practice. 3d. A systematic and successive order of studies for the student. 4th. A certain amount of direct clinical instruction in a public Hospital as a part of the senior course. The desirableness of these changes is too apparent to require either argument or illustration. The plan for accomplishing them, adopted by the convention, as expressed in the foregoing propositions, is simple and easy of execution, provided the several colleges will act in concert.

It requires each college to obtain and place on record sufficient evidence that every student admitted to matriculation possesses a certain amount of preliminary education. It requires *attendance* and *pay* for three annual courses of college instruction, as a condition for graduation; and arranges the whole curriculum of the college into three corresponding series of branches, so that each student can limit his attention to one series each year, thereby laying a foundation and building on it a superstructure in their natural order.

It contemplates such an increase in the number of members of each college Faculty, that four lectures per day can be given to each of the three *classes* in attendance, throughout the whole college term of six months. This would afford a very full course of instruction in each of the three *series* of *branches*, and yet give to the members of each class time fully to digest the instruction received. This would make it necessary during a part of each day that lectures should be given at the same hours to different classes. But as all Medical Colleges contain two and some of them three Lecture rooms, this would be attended by no inconvenience to the Faculty or students. The only valid objection which has been suggested by those connected with the Medical Colleges, is, that the increase in the number of each College Faculty required by the proposed plan, would necessitate a corresponding greater division of the income of each college, and thereby seriously reduce the amount received by any one member. If it is remembered, however, that while the plan requires a moderate addition to the number of members in the Faculties of most of the colleges, it also requires each student to attend and *pay full fees* for three courses of instruction instead of two, it will be seen that the revenues of each college derived from Lecture fees, will be increased in full proportion to the increase of the Faculty. As most of the colleges have allowed each member of the Faculty to sell his ticket to the class and retain the proceeds as his individual compensation it has been thought, that the proposed division of the students, attending any given college, into three distinct classes, and assigning to each a distinct series of branches, would limit the sale of the tickets of any one professor to the special class receiving instruction in his department; and consequently would restrict his income in proportion to the restricted number of tickets sold.

This is simply a misapprehension. The Convention took no action regarding the rate of lecture fees in any of the colleges; but the plan proposed, was founded on the expectation that each student would pay the same aggregate fees annually, as under the old plan. For instance, a student attending his first course and taking out the four tickets of the Freshmen series, would pay the same amount for the four that he now pays for the seven or eight that cover the curriculum in most of the colleges at this time. Hence, although each member of the Faculty would sell a smaller number of tickets, the income from them would be nearly the same. Or each college could have all lecture fees, from the several divisions of the class, paid to a common treasurer, and each member of the Faculty allowed to draw on such treasurer for his proportion of the same.

The 4th section or proposition adopted by the Convention, was not designed to fix the titles to professorships in the colleges, but simply to designate what was deemed necessary to constitute a proper Medical College Curriculum, and to determine what part of that Curriculum should be included in each of the *three series* of studies. Uniformity among the colleges in regard to this division into series, is very desirable in order to enable students, if they choose, to attend one series of studies in one college and another series in another without confusion.

To obviate embarrassment in making the change from the present system of college instruction to the one proposed, we would suggest that all students who should have so nearly completed their period of study at the time fixed for making the change, that an attendance on a single additional course of Lectures would render them eligible to graduation should be allowed to complete their course by attending the *senior* department under the new arrangement; while all who are in the first half of their period of study, should be subject to the new arrangement in full.

That the interests of medical science, the honor of the profession, and the welfare of the people, urgently require important improvements in our system of medical education and medical college instruction, is apparent to all. The public sentiment of the profession as expressed through the National, State, and local Societies, and through the leading medical periodicals, cordially sanctions the plan here proposed. We therefore respectfully ask you to give it a full consideration, and return to the Chairman of the undersigned Committee answers to the following questions:

1st. Do your Faculty, together with the governing authority of your College, approve of the several propositions as a whole?

2d. If you do not approve of the plan of revision as a whole, what changes would you suggest?

3d. If you approve of the plan as a whole, or of all its essential features, will your College be ready to adopt it practically, and issue your Annual Announcement for the College term of 1868-9, in accordance therewith; provided all the principal Medical Colleges in this country (or at least those in the cities of Boston, New York, Philadelphia, Baltimore, Richmond, Charleston, New Orleans, Louisville, Cincinnati, St. Louis, Chicago, Buffalo, and Albany,) will agree to do the same at the same time?

The great desideratum is to secure both harmony and concert of action on the part of the Medical Colleges, in the adoption of such measures as will at once place the system of medical education in this country on such a basis as the extent of the science and the responsibilities of its practical application in the prevention and treatment of diseases, require.

N. S. DAVIS,<br>S. D. GROSS,<br>GEO. C. BLACKMAN,<br>F. DONALDSON, } Committee.

*Chicago, August 1, 1867.*

---

PRIZES OFFERED BY THE CONN. MEDICAL SOCIETY FOR 1868. —The undersigned, a Committe of the Connecticut Medica.

Society, offer, in behalf of said Society, the following Prizes, to be awarded in May, 1868, viz: They renew the offer of the Jewett Prize of Two Hundred Dollars, for the best essay on the question, "*By what hygienic means may the health of armies be best preserved?*"* They also offer the Russell Prize of Two Hundred Dollars, for the best essay on the subject "*The Therapeutic Uses and Abuses of Quinine and its Salts.*"

The offer of both these prizes is extended to all physicians and surgeons of the United States, and the British Provinces of North America. In awarding the Prizes, the Committee will feel authorized to regard the literary merits, as well as the professional and scientific value of the papers submitted; and should none be received which they think worthy of such generous prizes, they may take the liberty of witholding their decision until the offer can be renewed.

Competitors will send their essays, free of expense, to one of the Committee, on or before the first of March, 1868, each having on it a motto or device, which shall also be written or placed on a sealed envelope, inclosing the writer's name and address. The unsuccessful essays will remain with that member of the Committee in whose hands they were originally placed, subject to the order of their respective authors.

[It has been stated, that a degree of health which is unusual prevailed in the Union armies during the late war, and the mortallity from disease was much below the average in the great military campaigns of Europe. Is there truth in the statement? If true, to what extent is it so, and why is it? What are the facts? What is the explanation? These are transcendently important questions, to which the Committee can find no answer in the essays which have been offered for the Jewett prize. They would not presume to direct the inquiries of competitors, but in this way, (parenthetically, as it were,) suggest a topic for consideration.]

BENJAMIN H. CATLIN, M.D., of West Meriden,<br>

LEONARD J. SANFORD, M.D., of New Haven,<br>

HENRY BRONSON, M.D., of New Haven,<br>

MELANCTHON STORRS, M.D., of Hartford,<br>

CHARLES L. IVES, M.D., of New Haven,<br>

} *Committee.*

* Those who forwarded essays on this question last year, are requested to let them remain until a decision is made, or recall them, through a friend, for alteration.

---

New Mode of Treating Fracture of Lower Jaw.—Dr. Benj. H. Riggs, of Selma, Ala., reports in the *Southern Journal of Medical Sciences* for May, 1867, a case of compound fracture of the inferior maxilla, occurring in an adult, in the treatment of which he claims that he made use of a new method.

The point of fracture was "immediately to the right of the symphysis, and through the socket of the right central incisor, the outer half of that tooth being exposed to view; the right fragment was very much drawn inwards; the gum was freely severed at the seat of fracture." A pasteboard splint and Barton's bandage was first applied, and the patient put upon his good behavior. This method proved unsuccessful, for the patient took the liberty of neglecting the doctor's instructions as to rest and method of eating, so that at the end of six weeks there was found a fistula under the chin, but no evidence of an attempt at union of the bones. Dr. Riggs then determined to use some mechanical appliance within the mouth, and obtained the assistance of Dr. S. G. Todd, a dentist, who took an impression in soft wax, formed from it a plaster cast, and upon this constructed a silver band, which was "made to fit every inequality of the teeth, completely encircling the first nine; it was not quite as wide as the teeth were long, and did not interfere with the closure of the mouth. An intercommunicating portion extended from the front arm of the band to the posterior, at the seat of fracture." This appliance was found to maintain exact coaptation of the bones. A tin fracture-box, which was soon discarded as cumbersome and unnecessary, and a two-tailed bandage, completed the apparatus. Under this treatment the fistula closed at once, and the case seemed to progress favorably for some weeks; but at the end of that time it was found necessary to remove the right central incisor, which, being at the seat of fracture, had acted as a foreign body. It was then seen that there had been no attempt at bone formation. The band was now shortened and readjusted, after which the fistula again closed, and the case progressed favorably to the end of the treatment. At the end of six weeks from the extraction of the tooth, the apparatus was removed, and the patient discharged, although on account of the anæmic condition of his system, osseous union was not yet perfect.

---

Sulphite of Soda in the Treatment of Erysipelas.—Dr. Addinell Hewson says, he has obtained results from the use of sulphite of soda in the local treatment of erysipelas, which have been to him both interesting and surprising. In extensive trial of the remedy, both in hospital and private practice, he has never seen it fail, when thoroughly applied before the deep planes of cellular tissue had been invaded by the disease. Before such parts had become affected, a solution of ten grains of this salt to the ounce of water, when thoroughly applied on lint all over the surface affected, and to a considerable distance beyond it, and covered with oiled silk to prevent the evaporation of the solution, had not only produced a decided bleaching effect on the discolored surface in every such instance, in the first twenty-four hours of its use, but had invariably destroyed all traces of the disease in forty-eight hours from its first application. The effect was the same, whether the application was made in the traumatic or idiopathic form of the disease. He has thus cured twenty-seven cases, seven of which were of idiopathic erysipelas. Even in the cases where the deep planes of cellular tissue were involved, as well as the surface, the disease on the surface was always apparently affected by the application. It was most positively bleached in all instances, and in many was evidently destroyed, within the period above stated, even while that in the deeper part proceeded to suppuration.—*Trans. Col. Phys. Philadelphia.*

---

PHTHISIS IN BARBADOES.—Phthisis is a disease that has been supposed hardly to exist in the tropics, but observation of late years seem to say that such is far from being the case. In the island of Barbadoes it is much more common than formerly, and quite so amongst the negroes. Since the abolition of slavery, the diet of the blacks has probably been less nutritious than when they were not obliged to provide for themselves, consisting now chiefly of Indian meal, sweet potatoes, and flying fish—which last delicious fish is taken in immense numbers around the island; and to this cause the increase of disease seems to be attributed, though the general appearance of the blacks struck me as quite healthy. And I would remark that the number of mulattoes, in whom we generally find a greater tendency to tubercular affections than in the pure negroes, appeared to me to be small. I saw several cases of phthisis at the hospital, and was told that when patients come to the island with this disease no marked benefit is experienced, though the climate, if a tropical one could have any effect, seems to be all that could be desired. Scrofulous glands and disease of the joints are also common, but tubercular meningitis is very rare.—DR. J. B. S. JACKSON, *Boston Medical and Surgical Journal.*

---

THE FRENCH students pay for diplomas in medicine and surgery, $145; in natural sciences and mathematics, $100; and in chemistry and pharmacy, $60. The fees are much heavier in Italy, and generally less in Germany.

MORTALITY REPORT FOR THE MONTH OF AUGUST:—

Dr. Rauch, the Sanitary Superintendent, read the following Sanitary report for the month of August just closed:—

CAUSES OF DEATH.

| | | | |
|---|---|---|---|
| Accidents, | 18 | Gastritis, | 3 |
| Abscess, back of neck, | 1 | Gastro-Enteritis, | 1 |
| Apoplexy, | 3 | Heart Disease, | 7 |
| Atrophy, | 2 | Hemipligia, | 1 |
| Birth, premature | 28 | Hernia, inguinal | 1 |
| " still | 13 | Hernia, estrangulated | 1 |
| Brain Disease, | 1 | Hydrocephalus, | 10 |
| " Congestion of, | 5 | Hypertrophy of heart, | 2 |
| " Inflammation of, | 5 | Inanition, | 3 |
| Bright's Disease, | 1 | Inflammation, kidneys | 1 |
| Bronchitis, | 2 | Inflammation, tonsils | 1 |
| Bowels, inflammation of | 3 | Liver, cirrhosis of | 1 |
| " obstruction of | 1 | Liver, inflammation of | 1 |
| Cancer of face, | 1 | Lungs, congestion of | 2 |
| " " intestines, | 1 | Laryngetis, | 1 |
| " " liver, | 1 | Measles, | 17 |
| " " stomach, | 1 | Meningitis, | 7 |
| " " uterus, | 1 | Meningitis, Cerebro-Spinal, | 4 |
| Cirrhosis, | 1 | Meningitis, Tubercular | 2 |
| Chlorosis, | 1 | Nephritis, | 1 |
| Cerebritis, | 1 | Old Age, | 4 |
| Cholera-Morbus, | 7 | Ovarian, disease of | 1 |
| Cholera Infantum, | 212 | Paralysis, | 1 |
| Convulsions, | 28 | Peritonitis, | 1 |
| Debility, | 20 | Phlegmasia dolens, | 1 |
| Delirium Tremens, | 2 | Phthisis Pulmonalis, | 23 |
| Diarrhœa, | 45 | Pneumonia, | 4 |
| Diphtheria, | 4 | Purpura, | 1 |
| Dropsy, general | 7 | Rheumatism cardiac, | 1 |
| Dropsy, brain | 2 | Rheumatic gout, | 1 |
| Dropsy, chest | 1 | Scrofula, | 2 |
| Dropsy, heart | 1 | Small-Pox, | 11 |
| Dysentery, | 24 | Stomach, congestion of | 1 |
| Enteritis, | 10 | Suicide, | 2 |
| Entero-colitis, | 2 | Sunstroke, | 4 |
| Fever, Bilious | 1 | Softening of Spinal cord, | 1 |
| Fever, Congestive | 3 | Tabes Mesenterica, | 29 |
| Fever, Putrid | 1 | Tetaurus, | 1 |
| Fever, Puerperal, | 4 | Teething, | 40 |
| Fever, Scarlet, | 8 | Ulceration of Bowels, | 1 |
| Fever, Typhoid, | 13 | Whooping-Cough, | 6 |
| Fever, Typhus, | 5 | Unknown, | 3 |
| Total, | | | 697 |

NATIVITIES.

| | | | | | |
|---|---|---|---|---|---|
| Chicago, | 409 | Germany, | 68 | Prussia, | 3 |
| Other parts U. S., | 87 | Holstein, | 1 | Denmark, | 2 |
| Norway, | 21 | Scotland, | 3 | France, | 2 |
| Ireland, | 39 | England, | 11 | Nova Scotia, | 1 |
| Holland, | 7 | Sweden, | 6 | Unknown, | 8 |
| Bohemia, | 18 | Canada, | 6 | | |
| Total, | | | | | 697 |

### MARRIED AND SINGLE.

The following table gives the relative number of deceased, married and single:—

Single,............612 | Married,.......... 85 | Total,............697

Ages of the Deceased.—Under 5 years, 497; over 5 and under 10 years, 18; over 10 and under 20, 23; over 20 and under 30, 33; over 30 and under 40, 27; over 40 and under 50, 28; over 50 and under 60, 13; over 60 and under 70, 14: over 70 and under 80, 9; over 80 and under 90, 4; still born and premature, 26; unknown, 5. Total, 697.

### DEATHS IN EACH WARD.

The following table gives the average of deaths in each ward, on the basis of the population of 1866:

| Ward. | Mortality. | Pop. in 1866. | One death in | Ward. | Mortality. | Pop. in 1866. | One death in |
|---|---|---|---|---|---|---|---|
| 1... | 8 | 9,648 | 1,206 | 13... | 36 | 8,188 | 227 4-9 |
| 2... | 24 | 12,985 | 541 1-24 | 14... | 44 | 12,108 | 275 2-11 |
| 3... | 37 | 15,738 | 422 24-37 | 15... | 57 | 15,716 | 275 41-57 |
| 4... | 33 | 10,884 | 329 27-33 | 16... | 38 | 14,912 | 392 8-19 |
| 5... | 44 | 9,410 | 213 33-44 | Unknown, | 11 | | |
| 6... | 60 | 10,580 | 176 26-60 | Lake View, | 2 | | |
| 7... | 90 | 18,755 | 208 35-90 | Lake Mich., | 1 | | |
| 8... | 46 | 10,429 | 226 33-46 | Mercy hos., | 1 | | |
| 9... | 34 | 13,940 | 381 6-34 | County hos., | 14 | | |
| 10... | 21 | 11,416 | 543 13-21 | Orph. asy., | 4 | | |
| 11... | 47 | 12,924 | 275 | | | | |
| 12... | 45 | 12,695 | 282 1-9 | Total, | 697 | | |

Dr. Rauch further stated that the health of the city at this present time, was very good, though the number of deaths had somewhat increased during the last few days, when compared with the earlier part of the week.

---

Sloughing Produced by Local Anæsthesia.—The *London Lancet*, says: "We examined, a few days since, in the Middlesex Hospital, a young woman whose case is of no little importance in reference to the question of local as against general anæsthesia for operations. Mr. Lawson had diagnosed the existence of an abscess behind the patient's breast, and as the pus was very deep, (under the pectoral muscle indeed) the refrigerator was used, paraffine ether being employed. Congelation was rapidly produced, and kept up for a few minutes. The result has been, that a portion of skin, about an inch by three-quarters of an inch, over the upper part of the breast, has sloughed, and its healing will necessarily be attended by an unseemly scar. The patient is a maid-servant; were she unfortunately a lady, the undress of the modern ball-room would be impracticable without revealing such a blemish as might seriously damage her value in the matrimonial market. The case is worth remembering when exposed parts of the body are to be operated upon.

Statistics of Cholera in Italy.—Some statistics of the cholera recently published in Italy, show that, during the eight months or more of its prevalence in united Italy, there were 32,577 persons attacked, of whom 12,901 died. In all cases the majority of persons were men, and the proportion of married persons over the unmarried is very decided. The poor who were attacked, numbered 15,467; but among the better classes, the ratio of mortality was far higher, in fact, 60 per cent. But few young children were attacked, and the susceptibility of the disease was most evident between the ages of twenty and thirty-five.

---

Curare in Epilepsy.—Dr. Benedikt informs the Vienna Medical Society that the subcutaneous injection of curare has a favorable influence over epileptic diseases. A man, 20 years of age, had had epilepsy since he was nine years old. During five months he was subjected to curare injections in hospital. For the last fifteen months he has had no return of the fits. Four similar cases, equally successful, were related by Dr. Benedikt. The injections were used three times a week, under the skin in the neck, an eighth of a grain being used at each operation.—*British Medical Journal.*

---

Money Receipts to Sept. 25th.—Drs. Wm. Martin, Chicago, $1.00; N. R. Weede, Young America, Ill., 3.00; A. C. Buffum, Honolulu, Sandwich Islands, 5.00; B. See, Marshall, Ill., 3.00; F. R. Payne, Marshall, Ill., 3.00.

---

To Physicians.—By request, Prof. Horatio R. Storer will deliver his second private course of twelve Lectures upon the Treatment of the Surgical Diseases of Women, during the first fortnight of December, at his rooms in Boston. Fee $50, and Diploma required to be shown.

Certificates of attendance upon the course just completed have been issued to the following gentlemen: Dr. C. M. Carleton, Norwich, Ct.; Daniel Mann, Pelham, N.H.; G. E. Bullard, Blackstone, Mass.; J. A. McDonough, Boston, Mass.; M. C. Talbott, Warren, Pa.; H. Gerould, Erie, Pa.; E. F. Upham, West Randolph, Vt.; W. L. Wells, Howall, Mich.; and W. A. I. Case, Hamilton, C.W.

*Hotel Pelham, Boston, 1st July, 1867.*

